# An evaluation of hemoglobin measurement tools and their accuracy and reliability when screening for child anemia in Rwanda: A randomized study

**DOI:** 10.1371/journal.pone.0187663

**Published:** 2018-01-04

**Authors:** Megan Parker, Zhen Han, Elizabeth Abu-Haydar, Eric Matsiko, Damien Iyakaremye, Lisine Tuyisenge, Amalia Magaret, Alexandre Lyambabaje

**Affiliations:** 1 Nutrition Innovation, PATH, Seattle, Washington, United States of America; 2 Devices and Tools, PATH, Seattle, Washington, United States of America; 3 Department of Human Nutrition and Dietetics, University of Rwanda, Kigali, Rwanda; 4 Department of Pediatrics, Centre Hospitalier Universitaire de Kigali, Kigali, Rwanda; 5 Departments of Lab Medicine and Biostatistics, University of Washington, Seattle, WA, United States of America; Emory University/Georgia Institute of Technology, UNITED STATES

## Abstract

Blood hemoglobin (Hb) is a common indicator for diagnosing anemia and is often determined through laboratory analysis of venous samples. One alternative to laboratory-based methods is the handheld HemoCue® Hb 201+ device, which requires a finger prick and wicking of blood into a pretreated cuvette for analysis. An alternative HemoCue® gravity method is being investigated for improved accuracy. Further, recent developments in noninvasive technologies could provide an accurate, rapid, safe, point-of-care option for hemoglobin estimation while addressing some limitations of current tools, but device performance must be assessed in low-resource settings. This study evaluated the performance of two HemoCue® Hb 201+ blood sampling methods and a noninvasive device (Pronto® with DCI-mini™ sensors) in a Rwandan pediatric clinic. Reference hemoglobin values were determined in 132 children 6 to 59 months of age by using a standard hematology analyzer (Sysmex KN21^TM^). Half were tested using the HemoCue® wicking method; half were tested using the HemoCue® gravity method; and 112 had successful hemoglobin readings with Pronto® DCI-mini™. Statistical analysis was used to assess the level of bias generated by each method and the key drivers of bias. The HemoCue® gravity method was the least biased. The HemoCue® wicking and Pronto® methods biases were inversely related to the Sysmex KN21^TM^ results. Both HemoCue® sampling methods correctly classified patients’ anemic status in 80% or more of instances, whereas the Pronto® device had a correct classification rate of only 69%. The HemoCue® gravity method was more accurate than the traditional HemoCue® wicking method in this study, but its accuracy and operational feasibility should be confirmed by future studies. The Pronto® DCI-mini™ devices showed considerable promise but require further improvements in sensitivity and specificity before wider adoption.

## Introduction

Anemia is one of the world’s most serious public health problems and is the second leading cause of disability [1]. Recent estimates by the World Health Organization (WHO) indicate that the highest prevalence of anemia is among children aged 9 to 59 months (42.6%) and pregnant women (38.2%) [2]. Africa is disproportionately affected, with 62.3% of preschool-age children and 46.3% of pregnant women suffering from anemia [2]. Rates of iron-deficiency anemia in Africa are lower than rates of overall anemia and are estimated at 20.3% and 20.2% for pregnant women and children under five years, respectively [3]. In Rwanda, nearly two in five children (37%) under five years of age have some level of anemia. Approximately 21% suffer from mild anemia (10.0–10.9 g/dl), 15% from moderate anemia (7.0–9.9 g/dl), and less than 1% from severe anemia (<7.0 g/dl) [4].

Hemoglobin (Hb) measurement is one of the most frequently ordered tests to detect anemia in both acute care and outpatient settings [5]. In well-resourced settings, Blood Hb concentration is measured routinely using a venous sample and large, expensive, stationary, and automated hematology analyzers (e.g., Sysmex KN21™). Although this method is accurate and reliable, it requires well-equipped laboratory facilities and trained personnel, which are often unavailable in resource-poor settings, especially in rural areas.

### HemoCue® analyzer

In settings where automated hematology analyzers are unavailable (e.g., small health clinics, community surveys), small mobile devices like the HemoCue® Hb 201+ are sometimes used. The standardized HemoCue® capillary blood collection method involves using a lancet to prick the finger and then directly wicking the blood into a microcuvette for analysis. Non-standardized capillary blood collection methods have also been observed in large-scale community surveys, whereby a lancet is used to prick the finger and milk blood onto an intermediate surface before it is taken up into a microcuvette. This non-standardized technique is known as the HemoCue® gravity method.

Although the accuracy and precision of HemoCue® 201+ for Hb measurement have been validated, its reliability depends on the quality of the blood samples. It is therefore important to understand the effects of different blood collection techniques on the accuracy of measurements. Previous studies comparing HemoCue® Hb measurements of capillary samples and results from an automated hematology analyzer using venous blood samples reported significantly higher Hb values for capillary samples from healthy blood donors (mean difference = +0.7g/dl) [6], pregnant women (mean difference = +0.8) [7], and anemic children (mean difference = +1.2) [8]. In the present study, the results obtained from two different HemoCue® blood collection techniques were assessed to determine ways to improve HemoCue® 201+ measurements, especially for infants and young children.

### Pronto® device

Hb assessments in infants and young children pose unique challenges in non-clinical settings because blood draws are difficult, invasive, painful, and sometimes unacceptable to parents. Noninvasive technologies that use a simple finger probe and are based on infrared spectrophotometry, using wavelengths like a conventional pulse oximeter, could provide alternative tools for Hb estimation. If shown to be accurate, these noninvasive devices could potentially offer a more acceptable, sustainable, and environmentally friendly alternative because they are pain-free and do not generate biohazardous waste.

The Pronto® device with DCI-mini™ sensors (Masimo Corporation, Irvine, CA) is designed for use with infants and children weighing 3 to 30 kg. The device has already been assessed in high-resource settings, primarily for continuous monitoring of pediatric patients in critical care settings [9, 10], but evidence to support its use in Africa and for community-based screening is not yet available.

### Aim of the study

The aim of this study was twofold: (1) to evaluate the accuracy of both the HemoCue® Hb 201+ wicking and gravity methodologies for measuring Hb levels and (2) to evaluate the accuracy of the Pronto® device with DCI-mini™ sensor for measuring Hb levels among children in Rwanda. All three methods were compared against the results with a standard reference hematology analyzer (Sysmex KN21^TM^). The investigators hypothesized that the two HemoCue® methodologies and the Pronto® with DCI-mini™ would approximate Hb values within ±1.0 g/dl of the reference values determined with the hematology analyzer. A second hypothesis was that the HemoCue® and Pronto® devices and would correctly classify patients’ anemic status, with confidence intervals for correct classification rates that excluded values lower than 65% to 69%, depending on observed biological variability. The results of this study will be used to inform health providers and key stakeholders about the accuracy of these devices and Hb measurement methodologies in resource-poor settings such as Rwanda.

## Methods

### Study design

This cross-sectional study was conducted at Centre Hospitalier Universitaire de Kigali (CHUK), the largest public referral and teaching hospital in Rwanda’s capital city between September and December of 2015 (weather temperature range: 15–30°C). Eligible participants were children 6 to 59 months of age who weighed 3 to 30 kg. Because the study included patients who were already required to provide a venous blood sample for their health care assessment, no additional venous blood samples were collected. To ensure a random sample was selected, every second eligible child was recruited and invited to enroll in the study by the study coordinator. The parents of each child provided informed consent prior to study participation.

**Fig 1:**
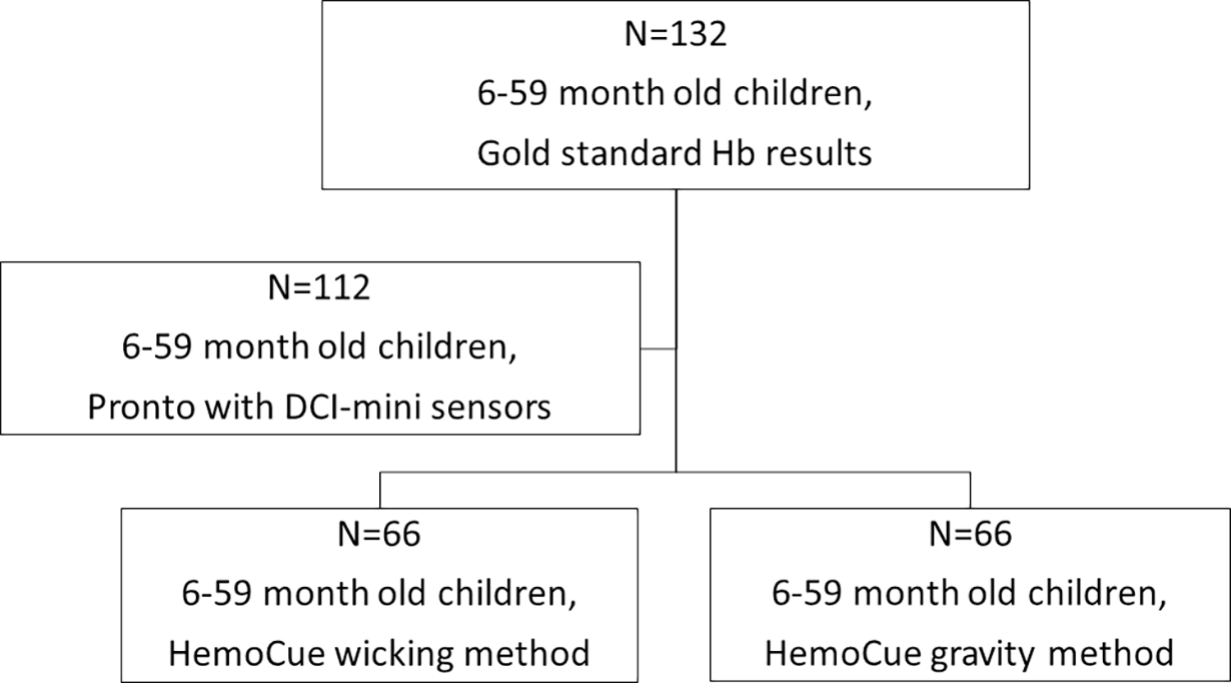
Study design.

At the time of venous blood sampling, two additional Hb measurements were conducted for each participating child by using the HemoCue® and Pronto® devices. Half of the 132 children were assessed with the HemoCue® wicking method, and the other half, the gravity method (Fig 1). For the Pronto® device measurements, two readings were required from each child, with a maximum of four attempts. If the first two readings were greater than 1.0 g/dl apart, a third reading was taken and averaged with the closer of the two previous values. If only one reading was obtained, the child was excluded from the analysis. Only 112 of the 132 children completed the required Pronto^®^ measurements to be included in the analysis (Fig 1). Fig 1 depicts the study design.

For each child, age, gender, height/length, Height for Age Z score (HAZ), Weight for Height Z score (WHZ), Mid-Upper Arm Circumference (MUAC), pigmentation, and other health information (such as recent morbidities and current medications) were recorded. The WHO Anthro software for PC was used to calculate Z scores (http://www.who.int/childgrowth/software/en/). Pulse pressure, profusion index, and functional oxygen saturation (SpO_2_) values that automatically appeared on the Pronto® device were recorded as well.

### Sample size

Simulations were used to assess the variability of the average difference between the HemoCue® or Pronto® with the venous blood draw (Sysmex KN21^TM^). Using standard deviations from previous studies conducted with children under five years of age in Guatemala, Tanzania, and Kenya, we simulated Hb measurements according to the reference standard with standard deviations between 1.3 g/dL and 1.6 g/dL [11, 12, 13]. To simulate the Hb level on the comparator method (HemoCue® or Pronto®), we assumed the values were correlated at between 0.80 and 0.95, and that the resulting value had the same variability as that under the Sysmex KN21^TM^. Datasets of various sample sizes between 25 and 150 persons were then created, and the confidence interval for the difference between the two measurements was computed. This process was repeated 1,600 times for each sample size, and the proportion of times that the 95% confidence interval for the difference in measurements did not include the difference of interest 1.0 g/dL was described as the power.

It was determined that we would need 100 child participants to complete the study for statistical power. With 100 samples and assuming the correlation (rho) between measurement methods is at least 0.93, we had 97–99% power to confirm that the average difference between the two Hb measurement methods did not exceed 1.0 g/dL. We anticipated a 10% study withdrawal or testing failure rate. To ensure at least 100 children completed the study, we chose a sample size of 132.

### Hemoglobin measurement devices

The HemoCue® Hb 201+ is a photometric device that uses specially designed microcuvettes containing three different dried reagents to estimate total blood Hb concentration. The blood sample is wicked into the pretreated cuvette from the fingertip. The Hb value is displayed on the device screen within 30 seconds. No dilution is required, and the device can be used with capillary, arterial, or venous blood.

The Pronto® DCI-mini™ is designed for use with infants and children weighing 3 to 30 kg. The DCI-mini™ sensor is a digital clip applied to a small child’s finger or on an infant’s big toe or thumb and uses a lightweight ribbon cable to connect to the Pronto® pulse oximetry device. By passing various wavelengths of visible and infrared light through a fingertip or toe, the detector within the sensor can measure changes in light absorption during the blood pulsatile cycle and send the signals to the Pronto® device. The device then uses a multi-wavelength calibration equation to quantify the percentage of total hemoglobin in blood. The Hb value in g/dl is displayed on a screen within 40 seconds. In addition, the device displays a measure of oxygen saturation (SpO2) and pulse rate.

### Statistical design and analysis

To validate and determine the accuracy of the Pronto® with DCI-mini™ sensor and the two HemoCue® Hb 201+ collection approaches, the results from these three methods were compared with the Hb values obtained by using a standard reference hematology analyzer (Sysmex KN21™). A Pearson’s correlation coefficient (r) was calculated to determine the overall level of agreement between the three methods and the standard reference Hb values.

The differences between the Hb values obtained from the three testing methods and the standard reference hematology analyzer were calculated for each child. The mean, standard deviation, 95% confidence intervals and ranges of the difference between methods (bias), and the 95% limits of agreement were calculated. Bland-Altman plots were created for each method as the correlation between the bias and the mean of the results and the Sysmex KN21^TM^ results. To investigate the factors contributing to the bias, linear regression was conducted between the bias and the standard reference hematology results, child’s age, child’s age squared, HAZ, WAZ, and MUAC to assess whether these factors influenced the correlation. Multivariate regressions were also conducted with these variables to assess the important factors that influence the biases.

Anemia classification rates were compared for the three testing methods relative to that of the Sysmex KN21^TM^ reference hematology, using 11.5 g/dl as the cutoff to adjust for the high altitude (1500 m) of Kigali. Sensitivity, specificity and overall correct classification frequencies are described for each POC test. All statistical analysis was conducted in STATA 12 software. A p-value less than 0.05 was considered statistically significant. The trial was registered on Clinicaltrials.gov (NCT02603250).

### Ethical approval and consent to participate

Ethical approval for this study was obtained from the PATH research ethics committee and the Rwanda National Ethics Committee.

## Results

### Demographics

The 132 study participants ranged in age from 6 to 59 months with an average weight of 10.7 kg (range 4.9 to 24.0 kg). Most participants had a Massey (skin pigmentation) score between 5 and 7 (80%), a HAZ score above –2 (81%), a WHZ score above –2 (77%), and a green MUAC score (83%). The demographic information did not appear to differ between the subsets of participants who contributed to the various study arms (Table 1).

**Table 1:** Demographic and physical characteristics of the sample population.

	Sysmex KN21^TM^ (n = 132)	HemoCue® wicking (n = 66)	HemoCue® gravity (n = 66)	Pronto® (n = 112)
**Gender**				
Male	58%	58%	58%	62%
**Weight (kg)**				
Mean (SD)	10.7 (3.4)	10.5 (3.8)	10.8 (2.9)	10.8 (3.4)
**Child rank**	2.2 (1.6)	2.1 (1.4)	2.2 (1.7)	2.2 (1.6)
**Age**				
6–8 months	18, 14%	10, 15%	8, 12%	15, 13%
9–11 months	21, 16%	10, 15%	11, 17%	18, 16%
12–17 months	28, 21%	16, 24%	12, 18%	24, 21%
18–23 months	13, 10%	5, 8%	8, 12%	9, 8%
24–35 months	24, 18%	13, 20%	11, 17%	21, 19%
36–59 months	28, 21%	12, 18%	16, 24%	26, 23%
**Skin pigmentation (Massey) scale**				
1	0, 0%	0, 0%	0, 0%	0, 0%
2	1, 1%	1, 2%	0, 0%	0, 0%
3	3, 2%	1, 2%	2, 3%	3, 3%
4	11, 8%	7, 11%	3, 5%	10, 9%
5	16, 12%	7, 11%	9, 14%	13, 12%
6	45, 34%	22, 33%	23, 35%	37, 33%
7	45, 34%	22, 33%	23, 35%	37, 33%
8	12, 9%	6, 9%	6, 9%	12, 11%
**HAZ**				
0 or higher	49, 37%	25, 38%	24, 36%	43, 38%
-2 to 0	58, 44%	30, 45%	28, 42%	51, 46%
-3 to -2	11, 8%	3, 5%	8, 12%	9, 8%
-3 or below	14, 11%	8, 12%	6, 9%	9, 8%
**WHZ**				
0 or higher	46, 35%	23, 35%	23, 35%	39, 35%
-2 to 0	55, 42%	28, 42%	27, 41%	46, 41%
-3 to -2	13, 10%	3, 5%	10 15%	12, 11%
-3 or below	18, 14%	12, 18%	6, 9%	15, 13%
**MUAC**				
Green (from 12.5)	110, 83%	53, 80%	57, 86%	96, 86%
Yellow (11.5–12.5cm)	13, 10%	6, 9%	7, 11%	12, 11%
Red (0–11.5cm)	9, 7%	7, 11%	2, 3%	4, 4%

### HemoCue® measurements

With the HemoCue® wicking method (WHb), the average Hb concentration measured was 11.6 g/dl, compared with 12.0 g/dl when the Sysmex KN21^TM^ test (LabHb) was used for the same population (Table 2). With the HemoCue® gravity method (GHb), the Hb measurements averaged 11.8 g/dl, compared with 11.7 g/dl with Sysmex KN21^TM^. The measurements from both HemoCue® methods were strongly correlated with their respective Sysmex KN21^TM^ results. The HemoCue® gravity and Sysmex methods, however, were more strongly correlated than were the HemoCue® wicking and Sysmex methods (HemoCue® wicking: r = 0.8, p<0.001; HemoCue® gravity method: r = 0.9, p<0.001). Most (91%) of the HemoCue® gravity method results fell within 1.0 g/dl of the Sysmex KN21^TM^ values; 7% were within 1.0 to 2.0 g/dl of the Sysmex values; and only 2% were more than 2.0 g/dl from the Sysmex values. In contrast, only 77% of the HemoCue® wicking method results fell within 1.0 g/dl of the Sysmex values, and 5% were more than 2.0 g/dl from the reference values.

**Table 2:** Blood hemoglobin concentrations of participant groups as determined by various methods.

Parameter	Testing method					
	Sysmex KN21^TM^	HemoCue® wicking	Sysmex KN21^TM^	HemoCue® gravity	Sysmex KN21^TM^	Pronto® DCI-mini™
	LabHb (n = 66)	WHb (n = 66)	LabHb (n = 66)	GHb (n = 66)	LabHb (n = 112)	PrHb (n = 112)
**Hb Mean (SD)**	12.0 (1.4)	11.6 (1.5)	11.7 (1.5)	11.8 (1.7)	11.9 (1.2)	11.6 (0.9)
**Hb Range**	8.4–16.2	7.9–16.1	7.3–19.5	6.5–19.4	7.3–15.0	9.7–14.3
**% Anemic**	36%	44%	38%	39%	34%	42%
**Pearson correlation**		WHb vs LabHb		GHb vs LabHb		PrHb vs LabHb
Estimate		0.8*		0.9*		0.43*
**Bias**		WHb-LabHb		GHb-LabHb		PrHb-LabHb
Mean (SD)		-0.3 (1.0)		0.0 (0.7)		-0.2 (1.1)
95% CI		-0.6 –-0.1		-0.1–0.2		-0.5–0.0
Range		-3.1–1.8		-2.9–1.7		-3.7–2.6
95% Limits of Agreement*		-2.3–1.7		-1.4–1.4		-2.4–2.0
**Absolute Difference (<1.0g/dl)**						
Mean (SD)		0.7 (0.7)		0.4 (0.5)		0.9 (0.7)
Range		0–3.1		0–2.9		0–3.7

* 95% Limits of Agreements were calculated as the mean bias ± 1.96 x SD

The average biases for the HemoCue® wicking and gravity methods were –0.3 and 0.0, respectively. Further, the 95% limits of agreements around the mean were wider for the HemoCue® wicking method than for the gravity method (Table 2). A Pearson correlation test indicated a negative relationship between the HemoCue® wicking method bias and the Sysmex KN21^TM^ Hb values (β = –0.21; 95% CI –0.38, –0.05) (Table 3). Further exploration indicated that the wicking method overestimated lower hemoglobin values (<11.5 g/dl) and underestimated higher hemoglobin values (≥ 11.5 g/dl) (data not shown). Interestingly, the HemoCue® gravity method did not appear to bias the results in any direction (β = 0.01; 95% CI: –0.10, 0.11) (Table 3).

**Table 3:** Factors influencing the bias of assessed testing methods, as evaluated by univariate and multiple regressions.

**Univariate regression**						
	**HemoCue**® **wicking method**		**HemoCue**® **gravity method**		**Pronto**® **device**	
**Variable**	**coefficient of variation**	**95% CI**	**coefficient of variation**	**95% CI**	**coefficient of variation**	**95% CI**
Sysmex KN21^TM^	-0.21*	-0.38, -0.05	0.01	-0.10, 0.11	-0.67*	-0.80, -0.54
MUAC	-0.09	-0.22, 0.03	0.02	-0.06, 0.11	-0.01	-0.14, 0.11
Childage	0.00	-0.01, 0.02	0.01	0.00, 0.02	0.00	-0.02. 0.01
Childage^2^	0.00	0.00, 0.00	0.00	0.00. 0.00	0.00	0.00. 0.00
HAZ	0.01	-0.12, 0.13	-0.02	-0.12, 0.08	0.03	-0.10, 0.15
WHZ	-0.11	-0.24, 0.02	-0.02	-0.10, 0.07	0.03	-0.09, 0.15
Skin pigmentation	—	—	—	—	-0.22*	-0.39, -0.05
SpO_2_	—	—	—	—	-0.02	-0.15, 0.11
Pulse rate	—	—	—	—	0.00	-0.01, 0.01
Perfusion index	—	—	—	—	0.01	-0.09, 0.12
**Multiple regression**						
	**HemoCue**® **wicking method**		**HemoCue**® **gravity method**		**Pronto**® **device**	
**Variable**	**coefficient of variation**	**95% CI**	**coefficient of variation**	**95% CI**	**coefficient of variation**	**95% CI**
Sysmex KN21^TM^	-0.24*	-0.43, -0.05	0.01	-0.09, 0.11	-0.66*	-0.79, -0.52
MUAC	-0.07	-0.33, 0.20	-0.02	-0.12, 0.08	0.01	-0.15, 0.18
Child age	0.01	-0.06, 0.08	0.02	-0.03, 0.06	0.03	-0.02, 0.08
Child age^2^	0.00	0.00, 0.00	0.00	0.00, 0.00	0.00	0.00. 0.00
HAZ	0.04	-0.14, 0.23	-0.02	-0.13, 0.09	0.01	-0.11, 0.12
WHZ	-0.09	-0.28, 0.11	0.01	-0.10, 0.12	-0.01	-0.15, 0.13
Skin pigmentation	—	—	—	—	-0.14	-0.29, 0.00
SpO_2_	—	—	—	—	0.02	-0.08, 0.11
Pulse rate	—	—	—	—	0.00	-0.01, 0.01
Perfusion index	—	—	—	—	0.00	-0.07, 0.08

*p<0.05

For both HemoCue® methods, univariate regressions were conducted to examine the relationship between the bias and the child size and pigmentation variables (child age, child age^2^, HAZ, WHZ, skin pigmentation). For the wicking method, the Sysmex KN21^TM^ measurement was the only factor that had a significant relationship with the bias. A multiple regression confirmed that the other variables could not explain further variation. For the gravity method, univariate regressions indicated that none of the variables had a significant relationship with the bias. A multiple regression confirmed this finding (Table 3). All variables were normally distributed except for child age.

### Pronto® DCI-mini™ measurements

Two successful Pronto® DCI-mini^TM^ readings were required for each of the 132 child participants within four attempts. Sixteen children were not able to have a Pronto® reading, and another four children had only one successful reading. Among the remaining 112 children (85%), 102 subjects had two readings within 1.0 g/dl in the first two attempts; seven required a third reading due to a difference greater than 1.0 g/dl between their first two readings; and three required four attempts to achieve two readings within 1.0 g/dl. Almost all measurements were taken using the thumb (n = 126; 95%); the remainder were taken using the ring finger (n = 1; 1%) or big toe (n = 5; 4%).

For children with Pronto® readings (PrHb), Hb concentrations averaged 11.6 g/dl, with a range of 9.7 to 14.3 g/dl (Table 2). The Sysmex test (LabHb) for the same population yielded a mean of 11.9 g/dl, with a range of 7.3 to 15.0. A Pearson’s correlation test indicated a significant positive correlation of moderate strength between the Pronto® and Sysmex results (r = 0.43; p<0.0001) (Table 2).

On average, the Pronto® device appeared to yield a slightly lower Hb value (bias = –0.2) than the Sysmex value, with a range of –3.7 to 2.6 and 95% limits of agreements of –2.4 to 2.0. Overall, 65% of the Pronto® Hb readings fell within 1.0 g/dl of the Sysmex values, and 91% fell within 2.0 g/dl. The investigators performed a univariate analysis to determine whether any of the variables influenced the level of bias. The variables investigated were related to the standard reference Hb value (Sysmex KN21^TM^), child size (HAZ, WHZ, MUAC), age, skin pigmentation, oxygen saturation (SpO_2_), pulse rate, and perfusion index. There was a strong, negative correlation between the Pronto® bias and the Sysmex KN21^TM^, indicating the Pronto® device overestimated lower hemoglobin values and underestimated higher hemoglobin values (β = –0.67; 95% CI: –0.80, –0.54). Univariate regressions also showed that the bias was negatively correlated with skin pigmentation (β = –0.22; 95% CI: –0.39, –0.05) (Table 3). When all of the variables were included in a multiple regression model, only the Sysmex KN21^TM^ remained significant.

Next, we evaluated the impact of each variable and its influence on the ability of the Pronto^®^ DCI-mini™ to generate readings. Participants who had no readings were more likely to have a lower MUAC value and lower functional oxygen saturation (SpO_2_) (Table 4).

**Table 4:** Factors influencing the ability of the Pronto® device to generate readings.

** **	Able to detect reading?		
	Yes	No	p-value
	Mean (SD)	Mean (SD)	
	(n = 112)	(n = 20)	
Age, year	23.3 (15.1)	21.5 (15.1)	0.62
Child birth rank	2 (2)	2 (1)	0.90
Height	83.1 (13.4)	78.3 (12.6)	0.14
HAZ	-0.4 (1.6)	-1.2 (1.7)	0.05
WHZ	-0.8 (1.7)	-0.8 (1.8)	0.95
**MUAC**	**14.8 (1.7)**	**13.2 (2.4)**	**0.00**
Skin pigmentation	6 (1)	6 (1)	0.68
Pulse rate	133 (19)	129 (28)	0.37
**SpO**_**2**_**%**	**96.7 (1.7)%**	**92.5 (8.2) %**	**0.00**
Perfusion Index	4.0 (2.0)	3.4 (2.0)	0.20
Hemoglobin	11.9 (1.2)	11.6 (2.7)	0.45

### Bland-Altman plots

**Fig 2:**
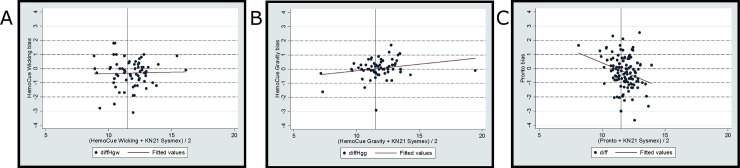
Bland-Altman plots for three hemoglobin measurement methods. Bland-Altman plots for all measurement methods. Y axes: bias produced by each method; X axes: the average of Hb results from each method and the Sysmex KN21. The three plots depict the HemoCue wicking method bias (A), HemoCue gravity method bias (B), and the Pronto device method bias (C).

The Bland-Altman plots (Fig 2) illustrate the association between the bias (difference in Hb measurement) and the average between each method and the Sysmex KN21^TM^ Hb value. For the HemoCue® wicking method, the inverse relationship described previously between the bias and the Sysmex KN21^TM^ (Table 3) was diluted when the bias was plotted against the *average* Hb result ((HemoCue® wicking Hb + Sysmex KN21^TM^ Hb)/2) and was no longer significant (r = 0.03, p>0.05). No statistically significant relationship was found for the HemoCue® gravity method. For the Pronto® DCI-mini™ method, the inverse relationship between the bias and the average Hb result ((Pronto® Hb + Sysmex KN21^TM^ Hb)/2) was significant (r = –0.3; p<0.01).

### Sensitivity and specificity

Relative to the Sysmex KN21^TM^ reference hematology analyzer, rates of correct classification of participants’ anemic status for both HemoCue® methods exceeded the anticipated range of at least 65–69% (HemoCue® wicking: 83%, 95% CI: 74% to 92%; and HemoCue® gravity, 80%, 95% CI: 70% to 90%). The rate of correct classification of participants’ anemic status for the Pronto® device, however, did not exceed the anticipated range of at least 65–69% (Pronto®: 69%, 95% CI: 60 to 78). Although the two HemoCue® methods showed higher sensitivity and specificity for correct classification than did the Pronto®-mini method in the study population as a whole, a re-examination of the data for children at least 18 months old found that the three methods gave comparable results (Table 5).

**Table 5:** Sensitivity, specificity, and correction classification rates for the three methods using a cutoff of 11.5 g/dl*.

	Sensitivity (%)	Specificity (%)	Correct classification (%)
**Children 6–59 months**			
HemoCue® wicking	88	81	83
HemoCue® gravity	76	83	80
Pronto® DCI-mini™	66	70	69
**Children 18–59 months**			
HemoCue® wicking (N = 30)	89	86	87
HemoCue® gravity (N = 31)	64	88	80
Pronto® DCI-mini™ (N = 65)	72	84	80

* 11.5g/dl is the anemia cutoff standard for Kigali (1500 m).

Unexpectedly, the HemoCue® wicking method appeared to have higher sensitivity rates than the HemoCue® gravity method. In this case, sensitivity is a measure of each test’s ability to indicate “anemia” among those who are anemic. Specificity indicates a tests’ ability to accurately classify people as negative who do not have anemia. Because most children in both subpopulations were not anemic, the heightened specificity of the gravity method may have helped it to give more accurate results.

## Discussion

Effective screening programs to assess the prevalence of anemia in vulnerable populations (e.g., women and children) are required for appropriate interventions to prevent and treat anemia. A reliable, sensitive, low-cost, safe, and rapid test for Hb measurement is urgently needed in low-resource settings. In this study, we assessed the accuracy of three different point-of-care (POC) approaches for Hb measurements compared to the reference Sysmex KN21^TM^ method used in Rwanda. To our knowledge, our study is the first to test the accuracy of Pronto^®^ DCI-mini™ in a developing-country setting.

Overall, the HemoCue® gravity method was the most accurate and least biased among this pediatric population. The HemoCue® wicking method and the Pronto® device were found to overestimate Hb concentration among anemic children and underestimate Hb concentration among healthy children. The accuracy of these two methods improved among children older than 18 months.

The HemoCue® wicking method is the approach recommended by the manufacturer, with specific instructions on proper methodology for blood sampling. Recent concerns about the accuracy and reliability of the method—thought to be caused by user variability and blood sampling techniques—has prompted an investigation into the use of the gravity method for blood sampling with the HemoCue®. As we found in this study, the HemoCue® gravity method appears to be more accurate than the HemoCue® wicking method. Additional studies with larger samples are needed to confirm this conclusion, and the mechanism by which enhanced accuracy is achieved warrants further investigation. It is possible that the transfer of blood samples onto a nonpermeable surface changes the physical properties of the blood collected. It is a limitation of this study that we did not do a similar comparison with venous blood to determine if contact with a nonpermeable surface prior to analysis in the Sysmex KN21^TM^ affected the results.

Although the HemoCue® gravity method provides potential improvement in measurement accuracy, it brings additional operational challenges, especially in low-resource settings. The use of HemoCue® 201+ in developing countries is already limited by the relatively high cost and limited availability of the disposable cuvettes. In addition, a clean surface is needed for the HemoCue® gravity method, which in turn introduces additional medical waste and risks for the staff conducting the tests. Moreover, the effects of user variability and environmental factors such as heat and humidity on the gravity sampling method have not been assessed. Future studies are needed to assess the operational feasibility of the gravity method.

Noninvasive measurement devices are promising tools that may have significant advantages over existing anemia screening methods. Devices such as the Pronto^®^ DCI-mini™ can be operated by minimally trained health workers and therefore increase opportunities for early identification and treatment of anemia.

Noninvasive measurements can also increase patient and caregiver acceptability, reduce the risk of infection from contact with blood and sharps, and reduce the burden on supply and distribution since no recurrent consumables are required [14]. However, our study indicated that the accuracy of the Pronto® device should be improved to fully realize its potential for POC, population screening or as a preliminary screening tool. The study did not assess the value of additional indicators provided by the Pronto® noninvasive device—SpO_2_ and pulse rate—which could potentially influence programs in their choice of methods. The benefits of noninvasive screening and diagnosis are clear, and it is anticipated that refinements to these innovations will continue to be made as user feedback and evidence on the performance of the devices is collected.

Our study of the Pronto^®^ DCI-mini™ indicated a detection failure rate of 15%, and failures were associated with lower MUAC and SpO_2_ values. This failure rate was higher than in previous studies with other Masimo devices (e.g., 8% with Pronto®-7 as reported in 2011 [15], 1.7% with Pronto®-7 as reported in 2012 [16], and 2.5% with Pronto®-SpHb [17]). Consistent with our findings, Gayat et al. [15] identified lower SpO_2_ as a factor contributing to detection failure (possibly indicating other morbidities), in addition to lower diastolic blood pressure, lower Hb values, and older ages. Although other studies have noted that participants with darker or calloused skin or in settings with high ambient light had higher rates of repeated measurements and device failures [18], this study did not support those findings. It is important to conduct future studies to confirm our results and further assess other factors potentially contributing to detection failure. It is worth noting that lower MUAC and SpO_2_ could happen more frequently in low-resource settings due to higher rates of malnutrition or comorbidities.

This study was limited in that it used a relatively small sample size within a controlled hospital setting. Regarding generalizability, more research needs to be conducted with larger sample sizes in more diverse environments to better understand how noninvasive Hb measurement devices in particular will be used, how well they perform under routine conditions, and the feasibility of integration into current public health programs in low- and middle-income countries. Further research is also needed to evaluate the acceptability and operational feasibility of the HemoCue® gravity method of blood sampling, with blood sampling carried out in less controlled primary health care settings or during routine population surveys. The ultimate goal is to identify the most accurate, acceptable and affordable Hb measurement device that will lead to increased anemia testing and treatment.

## Conclusions

This study found that the HemoCue® gravity method was more accurate than the traditional HemoCue® wicking method in this population and setting, but the gravity method’s accuracy and operational feasibility should be confirmed by future studies. In addition, the Pronto® DCI-mini™ devices showed considerable promise but require further improvements in sensitivity and specificity before wider adoption.

This study also documented the limitations of each of the three methods to measure blood Hb concentrations and identified some of the factors that could cause variability in Hb assessments. When making decisions about the most appropriate tool for anemia screening, anemia control programs must take into account and carefully weigh the tradeoffs in cost, safety, convenience, and accuracy.
